# Smoking During Pregnancy Increases Risks of Various Obstetric Complications: A Case-Cohort Study of the Japan Perinatal Registry Network Database

**DOI:** 10.2188/jea.JE20100092

**Published:** 2011-01-05

**Authors:** Kunihiko Hayashi, Yoshio Matsuda, Yayoi Kawamichi, Arihiro Shiozaki, Shigeru Saito

**Affiliations:** 1 Department of Basic Medical Sciences, School of Health Sciences, Faculty of Medicine, Gunma University, Gunma, Japan; 2 Department of Obstetrics and Gynecology, Tokyo Women’s Medical University, Tokyo, Japan; 3 Department of Obstetrics and Gynecology, Faculty of Medicine, University of Toyama, Toyama, Japan

**Keywords:** smoking during pregnancy, obstetric complications, perinatal epidemiology, case-cohort study, registry database

## Abstract

**Background:**

The adverse effects of maternal smoking on the health of pregnant women have been examined mostly on a disease-by-disease basis. The aims of this study were to evaluate simultaneously the effects of smoking during pregnancy on various obstetric complications, using data from a large medical database, and to investigate the expediency of using a case-cohort design for such an analysis.

**Methods:**

A case-cohort study was conducted within the Japan Perinatal Registry Network database. Perinatal information on infant deliveries was entered into the database at 125 medical centers in Japan. The base population of the study was 180 855 pregnant women registered in the database from 2001 through 2005. The outcome measures were the incidences of 11 different obstetric complications. Logistic regression models were used to estimate age-adjusted risk ratios (aRRs) and relative excess incidence proportions (REIs).

**Results:**

The overall prevalence of smoking during pregnancy was 5.8% in the base cohort, and the prevalence was higher among younger women. A comparison of the cases and control cohort showed that smokers during pregnancy had statistically significant higher risks for preterm rupture of the membrane (aRR: 1.67, 95% confidence interval [CI]: 1.43–1.96; REI: 40.2%, 95% CI: 29.9%–49.1%), chorioamnionitis (1.65, 1.36–2.00; 39.4%, 26.4%–50.0%), incompetent cervix (1.63, 1.35–1.96; 38.5%, 25.8%–49.1%), threatened premature delivery (1.38, 1.17–1.64; 27.7%, 14.5%–38.9%), placental abruption (1.37, 1.10–1.72; 27.1%, 8.8%–41.7%), and pregnancy-induced hypertension (1.20, 1.01–1.41; 16.4%, 1.2%–29.3%).

**Conclusions:**

Maternal smoking was associated with a number of obstetric complications. This highlights the importance of smoking cessation during pregnancy. In addition, case-cohort analysis proved useful in estimating RRs for multiple outcomes in a large database.

## INTRODUCTION

According to the Organisation for Economic Co-operation and Development (OECD), the prevalence of current smoking in adult women is 13% in Japan, 14% in the United States, 19% in Germany, and 20% in the United Kingdom.^1^ Although many pregnant women try to quit smoking, and some tobacco control programs have successfully reduced the proportion of smoking mothers,^2^ the prevalence of smoking during pregnancy is approximately 6.5% in Japan,^3^^,^^4^ 12% in the United States,^5^ 12% in Germany,^6^ and 15% in the United Kingdom.^7^

The adverse effects of maternal smoking during pregnancy on the health of a fetus are well known and include still births, fetal growth restriction, decreased infant birth weight, and neonatal death. In addition, pregnant women who smoke may themselves experience complications such as premature rupture of the amniotic membrane. However, there are no comprehensive evaluations of the adverse effects of smoking on the health of pregnant women, because many different obstetric complications are possible and most studies examine these complications individually.

A case-cohort study—which is identical to a case-base design in which the base cohort is closed and the measure of interest is an incidence proportion rather than an incidence rate—is a variation of the case-control design in which the controls are drawn from the entire base population, regardless of their disease status.^8^^–^^10^ These conditions for the base cohort in a case-cohort design are common in perinatal epidemiologic studies.^9^^,^^10^ We evaluated the adverse effects of smoking during pregnancy on 11 different obstetric complications, using a population-based database, and examined the expediency of using a case-cohort design for such a comprehensive evaluation.

## METHODS

### Study design and data source

We conducted a case-cohort study using the Japan Perinatal Registry Network database, which was started in 1974 and is managed by the Japan Society of Obstetrics and Gynecology. This database was converted to its present database structure in 2001. It includes all live and stillbirths at 125 medical centers in Japan, including 76 university hospitals, 14 national hospitals, 10 Japanese Red Cross hospitals, and 25 other hospitals, and covered 5.2% (56 671 registered births) of the total 1 094 434 live and stillbirths in Japan in 2005.

A detailed description of the database has been published elsewhere.^11^ In brief, a self-administered questionnaire, interview, and medical records were used to collect information on maternal age, parity, cigarette smoking during pregnancy, alcohol intake during pregnancy, medical history, history of treatment for infertility, major obstetric complications during pregnancy, mode of delivery, and neonatal outcomes. Data entry was routinely performed by attendants at the time of delivery. The data conform to uniform coding specifications and diagnostic criteria for complications and were subject to rigorous quality checking. Smoking during pregnancy and the incidence of each obstetric complication were coded as “yes” or “no” in the database. The dataset for the study was provided by the Japan Society of Obstetrics and Gynecology. The study protocol was reviewed and approved by the ethics committee of Tokyo Women’s Medical University.

The base cohort of the study consisted of 180 855 pregnant women carrying a singleton fetus who were registered in the database from 2001 through 2005. Complete information on obstetric complications and smoking status during pregnancy was present for all women included in the base cohort.

### Case identification and control selection

From the base cohort, the cases were independently identified for 11 obstetric complications: threatened premature delivery before 37 completed weeks of pregnancy, incompetent cervix, pregnancy-induced hypertension, eclampsia, pulmonary edema, placental abruption, placenta previa, preterm premature rupture of the membrane (PROM) before 37 completed weeks of pregnancy, chorioamnionitis, placenta accreta, and disseminated intravascular coagulation syndrome.

**Figure 1.**
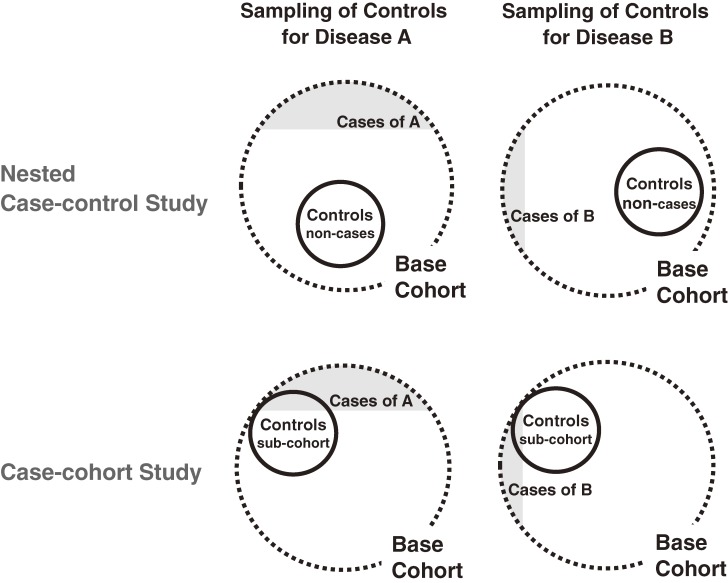
Control selection in nested case-control and case-cohort studies

The procedures for control selection in a conventional nested case-control study and a case-cohort study are illustrated schematically in Figure 1. To perform a comprehensive evaluation of multiple outcomes using a large database, a case-cohort study is preferable to a conventional nested case-control study, which is the most common study design.^10^ An advantage of the case-cohort design is that it allows the use of the same controls (ie, a subcohort selected from the base cohort) for several different outcome diseases. The control cohort in the current case-cohort study was selected randomly from the entire base cohort and included both cases and non-cases. We selected 3749 women for the control cohort, which represented at least 2% of all registered pregnant women in each hospital. The same control cohort was used in the analysis of each obstetric complication.

### Statistical analysis

Unconditional logistic regression models were used to estimate ratios of incidence proportions (risk ratios, RRs) and 95% confidence intervals (CIs) for the association of smoking during pregnancy with the incidence of each obstetric complication. The RR is estimated from the ratio of pseudo-risks by sampling controls from subjects at risk in a case-cohort design, and the incidence odds ratio (OR) is estimated by sampling from non-cases in a cumulative case-control study.^9^ We conducted a case-cohort comparison; therefore, logistic regression analysis provided an exact estimate of the RR without any arguments on a rare-disease assumption for outcome events. It is often assumed that the outcome disease under study is rare when the incidence OR approximates the RR. In general, this assumption is not needed, however, even in a case-control study. However, the incidence OR in a case-control study is not expected to be a good approximation of the RR, unless the incidence proportion is less than approximately 0.1.^9^

Smoking is one of the most preventable risk factors. For that reason, relative excess incidence proportions (REIs), which are identical to attributable fractions in the exposed population, and 95% CIs were calculated using the exact estimate of RRs:

$$\mathrm{REI}(\%)=\frac{\mathrm{RR}-1}{\mathrm{RR}}\times 100$$

The REI is the fraction of the obstetric complication burden among smokers during pregnancy that would not have occurred if the smokers had the same incidence of complications as nonsmokers during pregnancy.^12^

Wald’s χ^2^ test was performed in the logistic regression analysis, and *P* < 0.05 was considered statistically significant. All statistical data analyses were carried out using SAS ver. 9.1 (SAS Institute Inc., Cary, NC, USA).

## RESULTS

### Smoking during pregnancy in base cohort

The base cohort included 180 855 women. At the time of delivery, 2834 (1.6%) of the women were younger than 20 years, 17 867 (9.9%) were 20 to 24 years of age, 54 057 (29.9%) were 25 to 29 years of age, 67 886 (37.5%) were 30 to 34 years of age, 32 173 (17.8%) were 35 to 39 years of age, and 6038 (3.3%) were 40 years or older.

A total of 10 527 women (5.8%) in the base cohort smoked during pregnancy. The prevalence of smoking during pregnancy tended to gradually increase as maternal age decreased. In particular, the prevalence of smoking during pregnancy was high in women younger than 20 years (15.7%) and in women aged 20 to 24 years (10.7%), as shown in Table 1.

**Table 1.** Prevalence of smoking during pregnancy in base cohort

	Prevalence	No. of smokers		No. of women
All women	5.8%	10 527	/	180 855
Maternal age at delivery, years				
≤19	15.7%	444	/	2834
20–24	10.7%	1910	/	17 867
25–29	5.8%	3136	/	54 057
30–34	4.7%	3166	/	67 886
35–39	4.8%	1545	/	32 173
≥40	5.4%	326	/	6038

### Cases of obstetric complications identified in base cohort

In the base cohort, we identified 5681 cases (incidence proportion 3.14%) of threatened premature delivery before 37 completed weeks of pregnancy, 2943 cases (1.63%) of incompetent cervix, 7371 cases (4.08%) of pregnancy-induced hypertension, 143 cases (0.08%) of eclampsia, 76 cases (0.04%) of pulmonary edema, 1770 cases (0.98%) of placental abruption, 2369 cases (1.31%) of placenta previa, 6902 cases (3.82%) of preterm PROM before 37 completed weeks of pregnancy, 2508 cases (1.39%) of chorioamnionitis, 202 cases (0.11%) of placenta accreta, and 343 cases (0.19%) of disseminated intravascular coagulation syndrome.

### Effect of maternal smoking on obstetric complications

A total of 216 women (5.8%) in the control cohort smoked during pregnancy. The prevalence of smoking during pregnancy in the identified cases is shown in Table 2. The crude ratios of incidence proportions (crude RRs) of smoking were statistically significant for threatened premature delivery before 37 completed weeks of pregnancy, incompetent cervix, placental abruption, preterm PROM before 37 completed weeks of pregnancy, and chorioamnionitis. The estimates of ratios of incidence proportions adjusted by maternal age at delivery (age-adjusted RR) are also shown in Table 2. Maternal smoking during pregnancy was significantly associated with threatened premature delivery before 37 completed weeks of pregnancy (age-adjusted RR 1.38), incompetent cervix (1.63), pregnancy-induced hypertension (1.20), placental abruption (1.37), preterm PROM before 37 completed weeks of pregnancy (1.67), and chorioamnionitis (1.65).

**Table 2.** Prevalence of smoking during pregnancy, and risk ratios (RRs) and relative excess incidence proportions (REIs) for obstetric complications

	**Smoking** **prevalence**	**Crude RR** **(95% CI)**	**Age-adjusted RR** **(95% CI)**	**Age-adjusted REI^a^** **(95% CI)**
**Control cohort**	5.8%			
**Cases of obstetric complications**				
Threatened premature delivery^b^	8.0%	1.42 (1.20–1.68)	1.38 (1.17–1.64)	27.7% (14.5%–38.9%)
Incompetent cervix	8.8%	1.58 (1.31–1.90)	1.63 (1.35–1.96)	38.5% (25.8%–49.1%)
Pregnancy-induced hypertension	6.5%	1.14 (0.97–1.35)	1.20 (1.01–1.41)	16.4% (1.2%–29.3%)
Eclampsia	4.9%	0.84 (0.39–1.82)	0.82 (0.38–1.78)	
Pulmonary edema	6.6%	1.15 (0.46–2.88)	1.22 (0.49–3.06)	
Placental abruption	7.6%	1.34 (1.07–1.67)	1.37 (1.10–1.72)	27.1% (8.8%–41.7%)
Placenta previa	5.6%	0.97 (0.77–1.21)	1.07 (0.85–1.34)	
Preterm PROM^b^	9.3%	1.68 (1.43–1.97)	1.67 (1.43–1.96)	40.2% (29.9%–49.1%)
Chorioamnionitis	9.3%	1.68 (1.38–2.03)	1.65 (1.36–2.00)	39.4% (26.4%–50.0%)
Placenta accreta	7.9%	1.41 (0.83–2.39)	1.52 (0.89–2.59)	
DIC syndrome	7.3%	1.29 (0.84–1.98)	1.35 (0.88–2.08)	

CI: confidence interval; DIC: disseminated intravascular coagulation; PROM: premature rupture of the membranes.

^a^REI was calculated for obstetric complications significantly associated with maternal smoking during pregnancy.

^b^Before 37 weeks of pregnancy.

**Figure 2.**
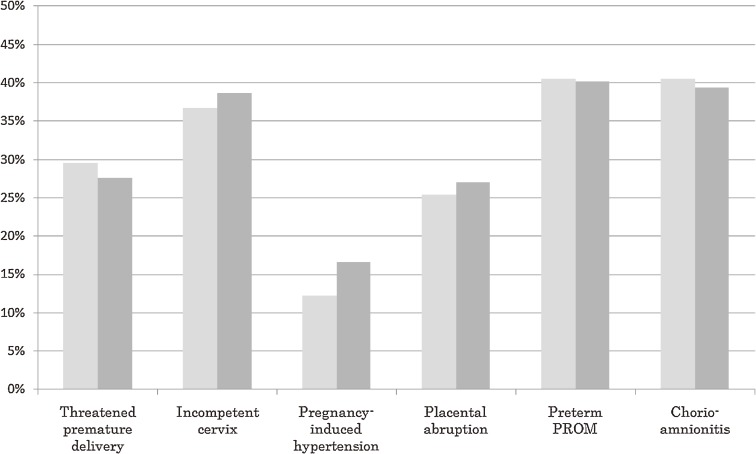
Relative excess incidence proportion (REI) of obstetric complications associated with smoking during pregnancy (left bars: crude REI, right bars: age-adjusted REI)

As for obstetric complications significantly associated with maternal smoking during pregnancy, crude and age-adjusted REIs are shown in Figure 2. The figure includes both crude and age-adjusted REI for pregnancy-induced hypertension, although the crude association was not statistically significant. Among smoking women, the age-adjusted REIs for threatened premature delivery before 37 completed weeks of pregnancy, incompetent cervix, pregnancy-induced hypertension, placental abruption, preterm PROM before 37 completed weeks of pregnancy, and chorioamnionitis were 27.7%, 38.5%, 16.4%, 27.1%, 40.2%, and 39.4%, respectively (Figure 2, Table 2).

## DISCUSSION

### Effects of maternal smoking on pregnancy-related complications

We found a statistically significant association between maternal smoking and the incidence of 6 of 11 obstetric complications. Obstetric complications may share many risk factors, of which maternal smoking appears to be one of the most relevant. Previous studies have reported similar adverse effects of maternal smoking on preterm PROM (OR 1.25 to 2.5),^13^^–^^15^ chorioamnionitis (bacterial vaginosis; OR 1.72),^16^ threatened premature delivery (OR 1.34 and 1.3),^13^^,^^17^ and placental abruption (OR 1.62 to 2.05).^15^^,^^18^^–^^20^ However, to the best of our knowledge, no other epidemiologic study has shown an association between maternal smoking and incompetent cervix, which was the fourth most common obstetric complication in the base cohort of the current study. This newly discovered association with maternal smoking deserves greater attention.

There is controversy regarding the effect of smoking on pregnancy-induced hypertension. Some studies have reported a large reduction in the risk of pregnancy-induced hypertension with maternal smoking (ORs of 0.6 and 0.80 for primiparous women^21^ and 0.81 for multiparous women)^22^; other studies have reported a statistically nonsignificant change (OR 1.1)^23^ or a strong positive association.^24^ We observed a slight increase in the risk of pregnancy-induced hypertension in smokers: the aRR was 1.20, which lies between the values noted in other studies. It is possible that the effect size in different studies varies according to other risk factors, such as chronic hypertension.

Eclampsia was the only obstetric event for which the risk was lower among women who smoked (aRR 0.82), although this decrease was not statistically significant. Other studies have also shown that smoking decreases the incidence of eclampsia (ORs of 0.7,^25^ and 0.74 for primiparous women and 0.75 for multiparous women).^22^

### Expediency of case-cohort design

The present case-cohort design was useful for comprehensively evaluating the risk of multiple outcomes associated with maternal smoking. Only 1 control subcohort was required for the analyses of 11 different obstetric complications. In contrast, if we had applied a nested case-control design, 11 different control groups would have been needed. Although we could have selected this 1 control group from subjects who were completely free from all 11 obstetric complications in a case-control study, this would have led to significant selection bias, as there are many common risk factors for the complications we studied. The subjects exposed to these common risk factors would have been systematically excluded if we had included only subjects free of complications. Such bias does not occur in a case-cohort design.

The case-cohort design was also advantageous in estimating REIs in the present study. The estimated RRs were relatively small: the largest aRR was 1.67 for preterm rupture of the membrane. REI is by definition more sensitive to a change in the estimated RR when the RR is closer to 1. In such a situation, the fact that case-cohort analyses provide an exact estimate of the RR, without the need for an approximation, made it possible for us to estimate accurate REIs.

### Methodological strengths and limitations of the study

Many studies have shown an effect of maternal smoking on a single obstetric complication. Such a disease-by-disease approach cannot distinguish between disease-specific variations and study-specific variations. However, the current case-cohort study revealed the adverse effects of maternal smoking on multiple complications.

This study does have some limitations. First, we could not examine the effect of smoking after adjusting for potential socioeconomic confounders because information on socioeconomic status was not available in the database. Second, smoking status during pregnancy was self-reported on questionnaires and in interviews. This type of information gathering likely underestimates the prevalence of pregnant smokers, because not all women will report their smoking^26^ and because smokers who quit during pregnancy tend to describe themselves as nonsmokers. This underestimation of smokers leads to potential underestimation of the effect size of smoking. Third, the database has no information on the number of cigarettes smoked daily. Therefore we could not examine any dose-response relationship between smoking and the incidences of obstetric complications.

### Conclusions

Maternal smoking was associated with a number of obstetric complications in a case-cohort analysis of a large perinatal registry database. The study highlighted the importance of smoking cessation during pregnancy. In addition, the case-cohort design proved useful in estimating relative risks for multiple outcomes in a large medical database.
